# Interaction with Blood Proteins of a Ruthenium(II) Nitrofuryl Semicarbazone Complex: Effect on the Antitumoral Activity

**DOI:** 10.3390/molecules24162861

**Published:** 2019-08-07

**Authors:** Bruno Demoro, Andreia Bento-Oliveira, Fernanda Marques, João Costa Pessoa, Lucía Otero, Dinorah Gambino, Rodrigo F. M. de Almeida, Ana Isabel Tomaz

**Affiliations:** 1 Cátedra de Química Inorgánica, Facultad de Química, Universidad de la República—UDELAR, 11800 Montevideo, Uruguay; 2 Centro de Química Estrutural, Faculdade de Ciências—CQE-FCUL, Universidade de Lisboa, 1749-016 Lisbon, Portugal; 3 Centro de Química e Bioquímica, Faculdade de Ciências da Universidade de Lisboa, 1749-016 Lisbon, Portugal; 4 Centro de Ciências Tecnológicas e Nucleares, Instituto Superior Técnico—C2TN-Técnico, Universidade de Lisboa, Campus Tecnológico e Nuclear, 2686-953 Sacavém, Portugal; 5 Centro de Química Estrutural, Departamento de Engenharia Química, Instituto Superior Técnico, Universidade de Lisboa, 1049-001 Lisbon, Portugal

**Keywords:** ruthenium(II), 5-nitrofurylsemicarbazone, cancer, cytotoxicity, plasma protein binding, fluorescence, circular dichroism, conditional binding constants, human serum albumin, human serum apo-transferrin.

## Abstract

The steady rise in the cancer burden and grim statistics set a vital need for new therapeutic solutions. Given their high efficiency, metallodrugs are quite appealing in cancer chemotherapy. This work examined the anticancer activity of an anti-trypanosomal ruthenium-based compound bearing the 5-nitrofuryl pharmacophore, [Ru^II^(dmso)_2_(5-nitro-2-furaldehyde semicarbazone)] (abbreviated as RuNTF; dmso is the dimethyl sulfoxide ligand). The cytotoxicity of RuNTF was evaluated in vitro against ovarian adenocarcinoma, hormone-dependent breast adenocarcinoma, prostate carcinoma (grade IV) and V79 lung fibroblasts human cells. The activity of RuNTF was similar to the benchmark metallodrug cisplatin for the breast line and inactive against the prostate line and lung fibroblasts. Given the known role of serum protein binding in drug bioavailability and the distribution via blood plasma, this study assessed the interaction of RuNTF with human serum albumin (HSA) by circular dichroism (CD) and fluorescence spectroscopy. The fluorescence emission quenching from the HSA-Trp214 residue and the lifetime data upon RuNTF binding evidenced the formation of a 1:1 {RuNTF-albumin} adduct with log *K*sv = (4.58 ± 0.01) and log *K*_B_ = (4.55 ± 0.01). This is supported by CD data with an induced CD broad band observed at ~450 nm even after short incubation times. Importantly, the binding to either HSA or human apo-transferrin is beneficial to the cytotoxicity of the complex towards human cancer cells by enhancing the cytotoxic activity of RuNTF.

## 1. Introduction

Cancer is the second largest cause of death in developed countries and Africa, Asia and Central and South America account for 70% of the world’s cancer deaths. According to World Health Organization, the number of worldwide deaths caused by this disease is projected to impressively increase in the next years, and it is expected that the annual cancer cases will rise up to 29.5 million within the next two decades [1,2]. GLOBOCAN statistics estimate that two in every five people will be diagnosed with cancer at some time before the age of 75, and one of them will effectively perish from that condition. The huge success of cisplatin (CisPt) and of other platinum anticancer drugs has broadened the field of cancer chemotherapy to include metal-based compounds [3,4,5] The outstanding efficiency of platinum-based drugs is well-known and although these metallodrugs are used in clinics as the first line treatment in over 50% of cancer cases, they show two main disadvantages which severely limit their clinical value: High toxicity with associated excruciating and debilitating side-effects that compromise the patient’s quality of life; and a high incidence of resistance, either acquired during the treatment cycles or intrinsic in non-responsive tumors [6,7,8,9]. Despite the huge efforts in research for the development of new antitumoral drugs that combine high efficiency with mild toxicity (desirably none), the quest for better therapeutic solutions continues. 

The overall activity of the metal-based compound is highly dependent both on the metal ion involved and on the structure of its ligands and the coordinating atoms that bind. Therefore, other non-platinum metal-based compounds have been widely investigated in the last years. In particular, ruthenium complexes have demonstrated a great potential for the development of promising drug candidates for cancer treatment and are now a recognized as effective alternatives in non-platinum drug development for cancer chemotherapy [9,10,11,12,13,14,15,16]. Indeed, Ru compounds have shown many chemical and biological advantages in respect of platinum-based antitumoral drugs affording different mechanisms of action, a lower toxicity and a different spectrum of activity as well as the potential to overcome platinum-resistance [7,16,17,18,19,20,21]. The promising drug candidates have evolved ranging from classical Ru(III) or Ru(II) complexes to organometallic piano-stool Ru(II) compounds [7,22,23,24,25,26,27,28,29,30] and several families in different development stages are reported to be interesting candidates. 

An appealing approach in metallodrug development is to combine pharmacophore moieties with biological activity in a chelating structure to bind the metal ion. Semicarbazones show a wide range of bioactivity, and their chemistry and pharmacological applications have been extensively investigated. These organic compounds and their metal complexes have shown a broad range of biological and therapeutic applications, like antiparasitic, anticonvulsant, anticancer and antimicrobial, among others [31].

**Figure 1:**
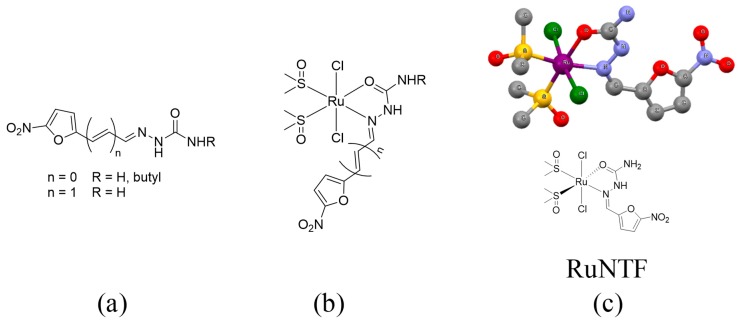
The structures of (**a**) anti-trypanosomic 5-nitrofurylsemicarbazone derivatives **L** and (**b**) their corresponding [Ru^II^Cl_2_(dmso)_2_L] complexes, including the (**c**) 5-nitro-2-furaldehyde semicarbazone ruthenium(II) complex (or RuNTF) studied in this work and the corresponding molecular structure as obtained from a single crystal X-ray diffraction study [33].

Some 5-nitrofurylsemicarbazone derivatives have shown activity against *Trypanosoma cruzi*, the parasite responsible for Chagas’ disease. In addition, a panel of [Ru^II^Cl_2_(dmso)_2_L] complexes (dmso is dimethyl sulfoxide, L is the semicarbazone) with these semicarbazone derivatives were previously developed by some of the authors as prospective antiparasitic compounds (Figure 1) and screened for their activity against *T. cruzi* [32,33]. 

The highly proliferative cells, such as tumor cells and trypanosomatid parasites, show many metabolic similarities that could lead to a correlation between antiparasitic and antitumor activities. In fact, several antitumoral drugs also show significant antiparasitic activity and vice-versa [34,35,36]. Although the antitumoral properties of these complexes have been preliminarily tested searching for a correlation of bioactivities, a more detailed examination of their potential in cancer therapy to afford a grounded conclusion about their eventual value is still lacking [37]. This work focuses on [Ru^II^Cl_2_(dmso)_2_(5-nitro-2-furyaldehyde semicarbazone)] (RuNTF in Figure 1c) to complete the previously reported studies. 

Understanding how potential drugs bind to serum proteins is of upmost importance in metallodrug development. The human serum albumin (HSA) is the most abundant protein in human plasma. It is the major nonspecific transporter in the blood and therefore plays a central role in drug distribution and pharmacokinetics [38]. Currently, the report on plasma protein binding is accepted as an FDA requirement for the early screening of a potential therapeutic agent [39]. 

The studies on the binding of a prospective drug to plasma proteins, in particular to HSA, are crucial for assessing its bioavailability. Indeed, albumin has a high impact on drug bioavailability since HSA binding can increase the solubility of a prospective drug, extend its in vivo half-life, increase the uptake of the drug by cancer cells, or in contrast have the opposite role, slowing down or preventing it reaching the target tissues [38,40,41,42]. Serum protein binding is thus recognized as a crucial factor in the in vivo performance of drugs [43]. It is particularly relevant for ruthenium-based agents, since albumin and transferrin are proposed to be relevant in their mechanism of action. Namely, albumin transporting and storing Ru-compounds, and transferrin providing active transport of these agents into the cells have been confirmed in several cases [16,19,39]. In addition, albumin plays a very important role by accumulating in malignant and inflamed tissue through the so-called enhanced permeability and retention effect. In this way, the interaction with HSA can afford additional selectivity by passively targeting the tumor tissue [43,44]. Interestingly, albumin nanoparticles of Paclitaxel, when intravenously administered, may improve the targeting variability by increasing the delivery to the cancer cells [45]. 

The authors have previously demonstrated that prospective anti-trypanosomal compounds [Ru_2_(*p*-cymene)_2_(L)_2_]X_2_, where X = Cl^−^ or PF_6_^−^ and L = deprotonated 5-nitrofuraldehyde thiosemicarbazones, also showed high antitumoral potential. Importantly, the presence of HSA in the incubation medium enhanced the cytotoxicity of the complexes, which correlated well with their HSA binding behavior [46].

This work re-evaluated the antiproliferative activity of the ruthenium compound [Ru^II^Cl_2_(dmso)_2_(5-nitro-2-furyaldehyde semicarbazone)], [Ru^II^Cl_2_(dmso)_2_(L)] or RuNTF, in three human cancer cell lines with different sensitivity to cisplatin (A2780 ovarian adenocarcinoma, MCF7 hormone-dependent breast adenocarcinoma and PC3 grade IV prostate carcinoma) at long incubation periods. The effect of serum protein binding on the cytotoxic activity using the ovarian A2780 cell line as a model has also been studied. As a first approach to the pharmacokinetics of RuNTF, this study assessed its interaction with HSA by circular dichroism (CD), UV-visible absorption and both steady-state and time-resolved fluorescence in simulated blood plasma conditions.

The quenching extent of the fluorescence emission from the HSA-Trp214 residue observed upon binding of RuNTF to HSA, indicates a strong complex-protein affinity, which is supported by CD data even for short incubation times. Importantly, the binding to serum proteins is beneficial to the cytotoxicity of the complex towards human cancer cells probably by enhancing the uptake of RuNTF into the cells. 

## 2. Results

The ruthenium complex RuNTF of formula [Ru^II^Cl_2_(dmso)_2_L] (where L = 5-nitro-2-furaldehyde semicarbazone) was synthesized for this work from the precursor [Ru^II^Cl_2_(dmso)_4_] with substitution of two dmso molecules by the semicarbazone ligand L, purified and isolated by this study as a pure dark red solid as described previously [33]. It was characterized by elemental analysis and spectroscopy, and the results were fully consistent with the formulation [Ru^II^Cl_2_(dmso)_2_(L)] for a mononuclear complex, with L behaving as a bidentate neutral chelator binding the metal ion in Z-conformation through the N_azomethynic_ and the O_carbonylic_ donor atoms in an octahedral arrangement. The coordination sphere was completed with two S-bound dmso molecules, and two *trans* chloride anions as co-ligands as depicted in Figure 1. 

The solution stability of RuNTF was studied before (at 28 °C, in 0.5% DMSO/100 mM phosphate buffer, pH = 7.4) [32,37]. Over time (till 48 h), the semicarbazone ligand was found to stay bound to the Ru(II) ion and an increase in the solution conductivity was observed, combined with a modest hypochromism (<10%) and a ~8 nm hypsochromic (blue) shift in the lower energy charge transfer band (~450 nm) of the electronic absorption spectrum. These results were interpreted as a consequence of a very slow single chloride substitution process by water. As such, this work used phosphate buffered saline (PBS) solution with a high chloride content to lessen Cl^−^ exchange processes, besides providing a suitable medium to mimic the blood plasma physiological conditions. 

Spectroscopic tools are frequently used to evaluate serum protein binding processes in solution media mimicking physiological conditions. The UV-Visible spectroscopy is a very convenient technique to monitor interaction processes of metal-ion complexes and it is a good tool as a first approach access to the binding of a metal ion compound to albumin. 

In the case of the HSA–RuNTF system, no changes were apparent in the isotropic absorption spectrum of the complex in the absence and presence of HSA, both at a reasonable (16 h) and at longer (96 h) incubation times (Figure S1). Thus, it can be concluded that the NTF ligand is maintained bound to Ru upon interaction with this protein. Besides this, the isotropic UV-Vis absorption does not provide much more relevant information for this system. In contrast, both circular dichroism spectroscopy and fluorescence emission spectroscopy proved to be fully adequate and invaluable tools to probe the HSA–RuNTF system. 

### 2.1. Interaction with Human Serum Albumin–Circular Dichroism Spectroscopy

Circular dichroism (CD) spectroscopy is a powerful technique especially suited for probing the binding of metal-based compounds to chiral macromolecules [47]. It is frequently used to monitor protein–compound interactions by following changes in the protein CD spectrum in the range 190–250 nm. Up to 250 nm, the protein exhibits a CD spectrum that reflects its secondary structure and provides reliable information on changes HSA eventually undergoes upon the binding of a compound. 

The CD spectrum of HSA has two negative bands in the far-UV range with maxima at 208 nm and 222 nm that are characteristic of an α–helical structure. For wavelength values above 300 nm, albumin does not absorb radiation. Nevertheless, CD can still be a very informative tool if an induced CD (ICD) spectrum is observed. An ICD band results from chirality that is transmitted to electronic transitions of an achiral compound if it binds in very close proximity to chiral groups of the protein [47]. The solutions of RuNTF in organic solvents or solutions of PBS buffer are CD silent since the Ru complex does not have stereogenic centres. However, RuNTF exhibits several absorption bands in the range 300–600 nm, where HSA does not absorb. The interaction of RuNTF with HSA can be confirmed if an ICD band is measured above 300 nm, since both the protein and complex are CD silent and only the protein-complex bound species may be detected. The observation of an ICD spectrum for λ > 300 nm would thus be a clear proof of the binding of RuNTF to the protein. 

#### 2.1.1. Time-Dependence 

**Figure 2:**
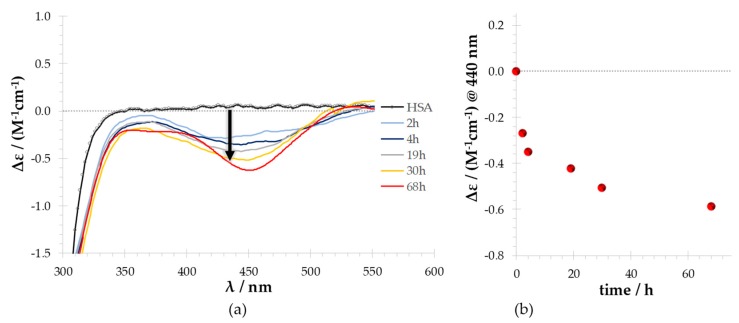
Time dependence for the RuNTF–HSA system at 1:2 molar ratio: (**a**) UV-Visible circular dichroism (CD) spectra of HSA (black line) and of solutions containing human serum albumin (HSA) and RuNTF (colored lines) measured over time; the arrow indicates an induced CD signal (ICD) (−) developing over time; (**b**) ICD(−) intensity measured at λ = 440 nm with increasing incubation time. [Conditions: C_HSA_= 100 µM and C_RuNTF_ = 200 µM; the samples incubated at 37 °C in 2%DMSO/PBS pH 7.4; the spectra recorded at room temperature (20.0 ± 0.5) °C.].

Time dependence of this interaction was examined to determine the equilibrium conditions for the RuNTF–HSA system in PBS pH 7.4:DMSO (98:2) aqueous medium. Figure 2 depicts the CD spectrum recorded for the RuNTF–HSA system in the near UV-Visible range (300–600 nm) over time.

A negative ICD signal is clearly detected as a broad band at ~440 nm. The observation of this ICD negative band (Δε < 0) indicates that RuNTF is located close enough to the protein to induce a chiral perturbation to its structure or electron rearrangements, with this chirality being transmitted to the charge transfer transition at 437 nm in the isotropic absorption spectrum making it CD active. The ICD(−) is clearly detected at an incubation time as short as 2 h, and becomes more intense as the incubation time is extended up to 68 h, with a ca. 100% increase in the Δε value measured. With the solutions containing HSA and NTF (Figure S2), no ICD bands were measured, thus indicating that the band at ca. 440 nm is due to the binding of the Ru center to a donor atom of the protein, such as a N_imidazole_ from a histidine residue (His242, a solvent accessible very likely candidate) or an NH_2_ donor from a lysine residue (e.g., Lys195, Lys199) [41], probably by the substitution of a Cl^−^ anion in the coordination sphere of RuNTF. 

The effect of RuNTF binding in the secondary structure of HSA was examined over time as well. Figure S3 collects the far UV–CD spectrum of the protein in the absence and in the presence of a 4-fold excess of RuNTF complex, confirming that α-helix content has not significantly changed. 

Thus, the time-dependent CD results indicate that RuNTF binds to human serum albumin without significantly affecting its secondary structure and, although a long contact time is required to attain true equilibrium conditions (≥62 h), a 2 h incubation period is enough to afford a significant amount of the RuNTF–HSA adduct. 

#### 2.1.2. Interaction in Equilibrium Conditions 

From the results presented above (Figure 2), it is clear that the main spectral changes observed for a 1:2 protein:RuNTF molar ratio on the ICD(−) bands were the increase in intensity of the band at ~440 nm. Based on that, this study chose two time points to evaluate this system with RuNTF:protein ratios ranging from 1:1 up to 1:10. 

**Figure 3:**
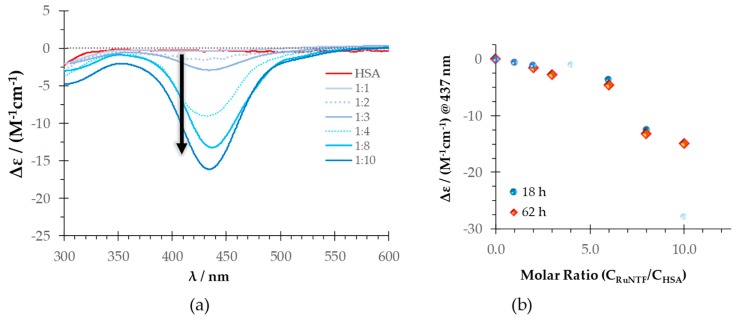
The induced CD band observed in the system RuNTF-HSA: (**a**) UV-Visible CD spectrum of the solutions containing HSA with increasing concentrations of RuNTF (Protein-to-Complex molar ratios are indicated) upon 62 h incubation times at 37 °C; (**b**) the ICD intensity measured at λ^(−)^_max_ = 437 nm with two different incubation times. [C_HSA_ = 100 µM = constant; C_RuNTF_: 0–1000 µM; the samples in PBS pH 7.4:DMSO (98:2) incubated for 18 h or 62 h at 37 °C; the CD spectra were recorded at room temperature (20.0 ± 0.5) °C immediately after each incubation period.].

Figure 3a shows the ICD spectrum recorded for the RuNTF–HSA system upon increasing the complex concentration after a 62 h incubation time. As before, increasing C_RuNTF_ leads just to an increase in the intensity of the ICD(−) band indicating that a higher concentration of the RuNTF–HSA adduct is present in the solution. When comparing the extent of the binding process at the two incubation times examined (Figure 3b), no major differences are found in the ICD(−) signal recorded, an exception made for the highest RuNTF concentration tested. In the latter case, Δε(−) measured at λ^(−)^_max_ = 437 nm at 18 h was much higher than the value obtained after a 62 h contact time. The very high concentrations needed for the ICD spectra, namely 1 mM RuNTF for the 1:10 molar ratio point (with HSA concentration of 100 μM) and the long incubation time might be causing the denaturation of albumin to some extent, yielding this somewhat surprising result. In contrast, far-UV CD spectra were obtained from the solutions with C_HSA_ = 5 μM (with C_RuNTF_ = 50 μM for the highest complex concentration used). 

Figure S4 depicts the far-UV CD signal recorded for HSA when titrated with RuNTF. Again, a small decrease in the spectrum intensity in the presence of RuNTF was observed that slightly reflects the increase in the concentration, but no difference in the overall protein secondary structure was detected.

As NTF alone, upon being incubated with HSA does not yield measurable ICD bands (see Figure S2) and, as confirmed by the UV-Vis data, the NTF ligand maintains its binding to the Ru center. The observation of strong CD bands in the interaction of RuNTF with HSA can only be explained by coordinative binding of Ru to the donor atoms of chiral residues of the protein. In this process, at least one of the labile monodentated donor ligands is removed. 

Taken together, the CD data clearly show that RuNTF binds to HSA and the ICD(−) spectrum observed indicates the formation of a stable RuNTF–HSA adduct, while not particularly affecting the protein secondary structure. Together with the absence of changes apart from the intensity, these results suggest that RuNTF is probably occupying a single predefined available location in the protein such as Sudlow’s drug binding site I or II (see below) [38,41] The ICD band observed upon the binding of RuNTF to HSA suggests the involvement of the protein residues in this process with the likely replacement of a labile ligand of RuNTF by e.g., the basic N_imidazole_ donor of His242 [41,48,49].

### 2.2. Binding Interaction with Human Serum Albumin–Fluorescence Spectroscopy

To gain further insight into the interaction between RuNTF and serum albumin, fluorescence spectroscopy was used, taking advantage of the intrinsic fluorescence of human serum albumin. HSA exhibits intrinsic fluorescence due to the presence of its phenylalanine, tyrosine and tryptophan residues, of which Trp is the dominant intrinsic fluorophore [50]. In addition, the high sensitivity of tryptophan to its local environment is a valued feature of intrinsic protein fluorescence. The two major structurally selective binding sites (Sudlow’s binding sites I and II) are typically considered for HSA [38,39,41]. HSA has 18 tyrosine residues and one sole tryptophan residue, Trp214, and its position in the protein is well defined: Trp214 is located in the protein subdomain IIA, near Sudlow’s drug binding site I but it is so sensitive to changes in its environment, either due to drug binding or due to structural alterations of the protein, that it can probe changes occurring in drug binding site II as well [51]. Trp214 can be selectively excited at 295 nm, thus becoming an intrinsic fluorescent structural probe in HSA. 

#### 2.2.1. Steady-State and Time-Resolved Fluorescence Emission

**Figure 4:**
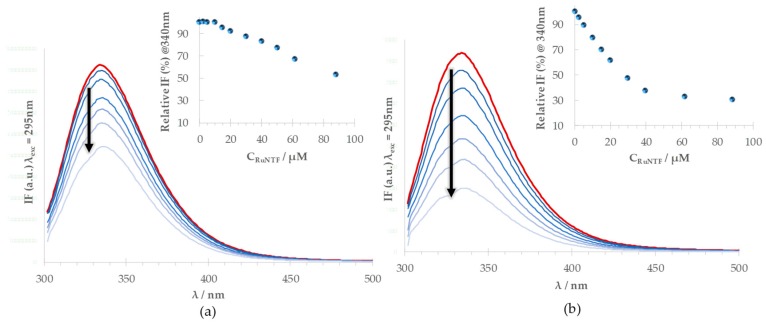
The effect of the RuNTF complex in the fluorescence emission of HSA-Trp214: Emission spectra of HSA in the absence (red, top line) and in the presence (blue) of increasing concentrations of RuNTF after an incubation time of (**a**) 16 h and (**b**) 96 h (arrows indicate the change with increasing complex concentration). **Insets**: The relative fluorescence intensity (%) at λ_em_ = 350 nm with increasing complex concentrations at 16 h (**a**) and 96 h (**b**). [Conditions: PBS pH 7.4/2% (*v/v*) DMSO; C_HSA_ = 5 µM, kept constant; λ_exc_ = 295 nm; the samples incubated at 37 °C; the spectra were recorded at (25.0 ± 0.1) °C.

The emission spectrum of the HSA-Trp214, selectively excited at 295 nm, in the presence of increasing concentrations of RuNTF is depicted in Figure 4. In the absence of the complex, the maximum emission intensity for Trp214 was observed at 334 nm (red line in Figure 4), since it is protected from the aqueous solvent, where free tryptophan would have a maximum emission at approximately 350 nm [52]. Considering the results presented above, the two different incubation times were assayed: 16 h and 96 h. In either case, there is a marked decrease in the Trp214 fluorescence intensity with increasing concentrations of RuNTF.

The strong quenching observed (the emission intensity reaches approximately 50% of the value in the absence of RuNTF after 16 h at the highest concentration of complex tested) indicates that there is a strong interaction between the protein and the Ru complex. This quenching is enhanced for the longer incubation time, with a further decrease in intensity to 30% after a 96 h contact time. A comparison of the relative intensity profile in the two cases (insets in Figure 4) shows that at 96 h, the interaction has reached completion.

**Figure 5:**
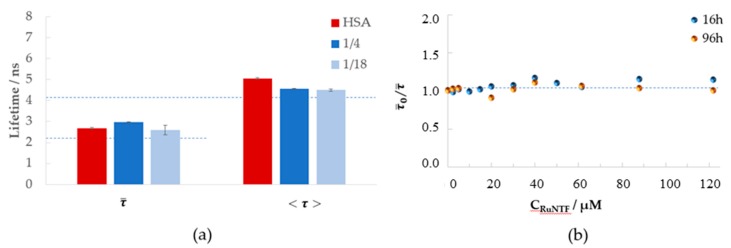
The effect of RuNTF in the fluorescence emission lifetime of HSA-Trp214: (**a**) amplitude-weighted ($\bar{\tau }$) and intensity-weighted (<τ>, see Equation (6) in Materials and Methods) the mean fluorescence lifetime, in the absence (red bars) and in the presence (blue bars) of RuNTF at the molar ratios [HSA]/[complex] indicated; (**b**) the amplitude-weighted mean fluorescence lifetime (see Equation (5) in Materials and Methods) in the absence ($\bar{\tau_{0}}$ ) and presence ($\bar{\tau }$ ) of the complex at the incubation time indicated [Conditions: PBS pH 7.4–2% (*v/v*) DMSO; C_HSA_ = 5 µM, kept constant; λ_exc_ = 279 nm, λ_exc_ = 340 nm; the samples were incubated for 96 h at 37 °C; the spectra were recorded at (25.0 ± 0.1) °C.]

In addition to the steady-state measurements, the time-resolved fluorescence experiments were also carried out. The fluorescence intensity decay of the Trp214 residue with time was measured for all samples as well. These two types of measurements are complementary and allow the differentiation of multiple binding modes near Trp214 and to clarify the quenching mechanism. Figure 5 depicts the amplitude-weighted mean fluorescence lifetime $\bar{\tau }$ (obtained from the intensity decays and calculated with Equation (6)—see Materials and Methods). 

Interestingly, no clear trend could be observed, and no significant variations in the fluorescence lifetime of the protein could be detected even at the highest complex concentration tested (122 µM) as compared to the fluorescence lifetime in the control, either at the shorter (16 h) or longer (96 h) incubation times. The amplitude-weighted mean fluorescence lifetime $\bar{\tau }$ remains unchanged in the presence and absence of the complex. This invariance strongly suggests that the compound is binding the protein in a very close vicinity (within molecular distance) of the Trp214 residue and implicates a mechanism of static quenching. 

#### 2.2.2. Quantifying the Interaction: RuNTF-HSA Binding Constant 

The results presented in Figure 4 and Figure 5 show that the fluorescence intensity of HSA-Trp214 markedly decreases upon the addition of the metal complex to the solution containing HSA, while the fluorescence lifetimes are unaffected.

**Figure 6:**
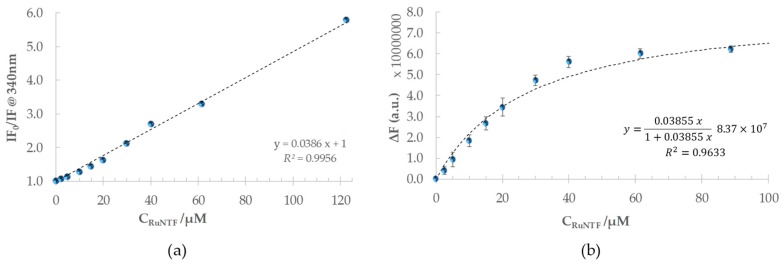
HSA-Trp214 Fluorescence intensity quenching with increasing complex concentration upon binding of RuNTF to HSA: (**a**) Stern-Volmer plot at 340 nm obtained from steady-state measurements (corrected for inner filter and self-absorption effects). (**b**) The data fit to the binding model according to Equation (3) (see text for details; ΔF = I_F(free)_ − I_F(bound)_ = I_F(HSA)_ − I_F_) [Conditions: PBS pH 7.4/2% (*v/v*) DMSO; C_HSA_= 5 µM, kept constant; λ_exc_ = 279 nm, λ_exc_ = 340 nm; the samples incubated for 96 h at 37 °C; the spectra recorded at (25.0 ± 0.1) °C].

The Stern–Volmer plots provide information on the mechanism causing the quenching observed and are depicted in Figure 5b ($\bar{\tau_{0}}/\bar{\tau }$ from time-resolved fluorescence data) and Figure 6a (corrected I_F0_/I_F_ data from steady-state results).

The linearity observed in the Stern-Volmer plot for Trp214 fluorescence quenching confirms that the binding of RuNTF to the protein proceeds through one sole interaction mode that strongly affects the Trp214 emission. This is fully consistent with the formation of a non-fluorescent 1:1 adduct in the ground state [53] with a conditional binding constant K’_B_ according to the equilibrium:

{HSA}_(aq)_ + {RuNTF}_(aq)_ ⇄ {HSA–RuNTF}_(aq)_ (1)

In such a case, Equation (2) describes the variation of I_F0_/I_F_ with C_RuNTF_,

(2) $$\frac{I_{F0}}{I_{F}}=1+\;K_{B}\;\;C_{RuNTF}$$

and the slope of the linear fit from the Stern–Volmer plot can be interpreted as a thermodynamic binding constant for this interaction [53], thus:

*K*_SV_ = *K*’_B_ = (3.855 ± 0.001) × 10^4^ M^−1^ or log *K*sv= 4.58 ± 0.01, and *K*_D_ = 25.9 μM

where *K*_D_ is the dissociation constant for the {HSA–RuNTF} adduct (or 1/*K*’_B_).

Figure 6b includes an alternative representation of the fluorescence quenching data, which derives from a model that describes well this 1:1 binding process but does not require the inversion of I_F_ values [51]. This model is valid for low complex concentrations or in systems where the interaction between the protein and the binding complex allows for the approximation C_Complex_−[{HSA−C}] ≈ C_Complex_, and is described by the hyperbole Equation (3):

(3) $$\Delta F=\;\frac{K_{B}\;C_{Complex}}{1+\;K_{B}\;C_{Complex}}\;\Delta F_{max}$$

where $\Delta F=\left(I_{F}^{free}-\;I_{F}^{bound}\right)=\;I_{F_{0}}-\;I_{F}$. Thus, *K*_B_ and Δ*F*_max_ can be retrieved from a non-linear fit to a plot of experimental ΔF values as a function of C_Complex_, as shown in Figure 6b. In the present case, Δ*F*_max_ was always equal or slightly higher than the initial fluorescence intensity, and therefore it was fixed to that value (Δ*F*_max_ = $8.37\times 10^{7}$) yielding:

$$\Delta F=\;\frac{0.03855\;\;C_{Complex}}{1+0.03855\;\;C_{Complex}}\;\left(8.37\;\times 10^{7}\right)\;\;\;\;\left(R^{2}=0.9633\right)$$

and *K*_B_ = (3.556 ± 0.005)$\times 10^{4}$ M^−1^ or log *K*_B_ = 4.55 ± 0.01. 

The *K*_B_ value calculated using this model (log *K*_B_ = 4.55 ± 0.01) is very similar to the binding constant obtained before from the Stern-Volmer plot (log *K*sv= 4.58 ± 0.01) and allows the conclusion that the fluorescence intensity tends to zero for higher RuNTF concentrations, in agreement with a static fluorescence extinction process by molecular contact with Trp214. The great spectral overlap between the emission and absorption in this system precludes the collection of reliable experimental data until saturation conditions, because self-absorption and the inner filter effects become too important for higher Ru-complex concentrations. Nevertheless, the fit to Equation (3) allows the circumvention of this experimental limitation. 

Overall, the fluorescence spectroscopy results confirm all the findings from CD spectroscopy, also proving the formation of a RuNTF-HSA adduct. The steady-state and time-resolved measurements allow for the conclusion that it is a 1:1 adduct (non-fluorescent) and RuNTF is very likely binding HSA within Sudlow’s drug binding site I. The conditional stability constant calculated for the RuNTF-HSA adduct (log *K*’_B_ = 4.5) reveals a moderate to strong interaction.

### 2.3. Cell Studies–Cytotoxicity against Human Cancer Cell Lines

**Figure 7:**
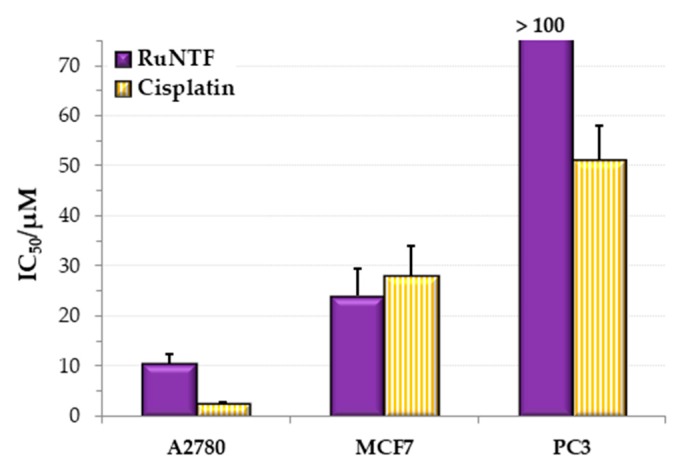
The cytotoxic activity of RuNTF expressed as the IC_50_ value after a 72 h challenge. (The results are average values from 2 independent experiments with a minimum of 6 replicates.).

The cytotoxic activity of RuNTF was evaluated against the representative human cell lines from common cancer diseases, namely A2780 ovarian carcinoma, MCF7 hormone-dependent breast adenocarcinoma and PC3 Grade IV prostate cells using the 3-(4,5-dimethylthiazolyl-2)-2,5-diphenyltetrazolium bromide (MTT) assay. The cell viability after 72 h incubation with the complex varied in a concentration-dependent mode, and the IC_50_ values, summarized in Figure 7, were obtained from the dose-response plots.

The cytotoxic activity against a non-tumoral cell line was also evaluated using the same experimental conditions and incubation time to have evidence of the selectivity of the complex for cancer cells (Figure S5). In the V79 lung fibroblasts, the IC_50_ value found was >200 µM which indicates no cytotoxic effect (in contrast with cisplatin that showed high cytotoxicity, with an IC_50_ value of 5.9 ± 0.9 µM). 

The cytotoxic activity of the complex is dependent on the nature of the cell line used. RuNTF exhibited good to moderate activity in the A2780 (cisplatin sensitive cells) and MCF7 cells but low activity for the highly metastatic PC3 cells. Interestingly, the results are related to the higher or lower sensitivity of these cells to the chemotherapeutic drug cisplatin [54] with RuNTF being more active against the CisPt sensitive cells.

### 2.4. Cell Studies–Effect of Serum Protein Binding on the Cytotoxicity 

To understand the mode of action of metal-based anticancer prospective drugs, the detailed study of their interactions with several biological targets is required, with plasma proteins being especially relevant. Albumin is the major non-specific carrier of endogenous and exogenous compounds in the human blood plasma. The interaction of albumin with metal anticancer drug candidates may have a major influence on the drug´s performance, resulting in drug deactivation or the formation of adducts that keep or enhance the initial drug efficacy. Transferrin (Tf) is also an important serum transport protein for other metal ions than iron. It has a substantially greater uptake into tumors, probably because of the large number of transferrin receptors in cancer cells. In addition, transferrin can act as a moderate selective carrier towards tumor cells. 

#### 2.4.1. Human Serum Albumin 

**Figure 8:**
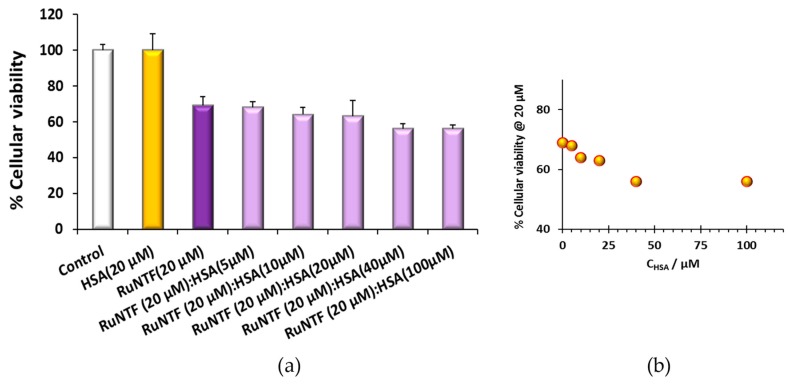
The effect of HSA on the cytotoxicity of RuNTF complex: (**a**) The cell viability (%) of RuNTF bound to HSA; (**b**) cytotoxic activity increase with HSA concentration (72 h challenge). The results are average values from 2 independent experiments with a minimum of 6 replicates; C_Complex_ = 20 μM.

Figure 8 depicts the results of the cytotoxic effect on the A2780 cells of RuNTF (20 µM) pre-incubated with HSA. The studies were conducted using different concentrations of HSA, and a control with HSA at 20 µM was made to evaluate its effect in the cells. Importantly, HSA binding enhanced the cytotoxicity of the complex when the protein concentration is high. The concentrations higher than 100 µM of HSA were not assayed due to the increased viscosity of the solutions and inherent foaming.

#### 2.4.2. Human Serum apo-Transferrin 

**Figure 9:**
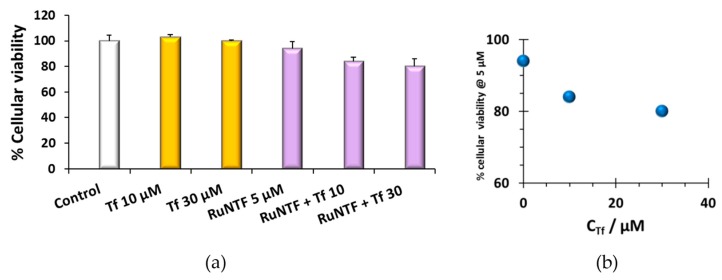
The effect of Tf on the cytotoxicity of the RuNTF complex: (**a**) The cell viability (%) of RuNTF bound to Tf; (**b**) cytotoxic activity increase with Tf concentration. (72 h challenge) (The results are average values from 2 independent experiments with a minimum of 6 replicates. C_Complex_= 5 μM).

Figure 9 shows the results of the cytotoxic effect of RuNFT in the presence of human serum apo-transferrin (Tf) at 10 µM and 30 µM in the A2780 cells after 72 h. The studies were conducted using a single concentration of RuNTF combined with the two different Tf concentrations. Similar to the effect of HSA, the pre-incubation of RuNTF with Tf enhanced the cytotoxicity of the complex in a dose-dependent way. 

## 3. Discussion

This work evaluated the potential anticancer properties for RuNTF against three human cancer cell lines with different response to the metallodrug cisplatin, namely A2780 ovarian adenocarcinoma, MCF7 hormone-dependent breast adenocarcinoma and PC3 grade IV prostate carcinoma.

The cytotoxicity found for RuNTF tested alone is dependent on the identity of the cell model used. From the dose-response cell viability plots, the following IC_50_ values (72 h) were calculated: (10.6 ± 1.9) μM for A2780; (24 ± 5.5) μM for MCF7; for PC3 IC_50_ > 100 μM, thus the complex is not active against this human cell line. In the A2780 cisplatin sensitive line, the Ru-complex exhibits an interesting IC_50_ value, although CisPt is ca. 4 times more cytotoxic for these cells [IC_50_(72 h, CisPt) ~ 2.5 μM]. The hormone-dependent breast adenocarcinoma line MCF7 is less responsive to CisPt [IC_50_(72 h, CisPt) ~ 30 μM] and the highly aggressive prostate PC3 is frequently considered resistant to CisPt [IC_50_(72 h, CisPt) ~ 55 μM)]. Thus, RuNTF exhibits in vitro a quite modest cytotoxicity against A2780, performing below CisPt for this cell line and also for PC3. It is, however, almost equally active than CisPt against MCF7 and not cytotoxic against the non-cancer lung fibroblast cells tested. This suggests an eventual intrinsic selectivity towards cancer cells that together with the cytotoxic activity shown (albeit modest) endows RuNTF with renewed value.

This study also focused attention on the role of the serum protein binding on the prospective anti-tumor activity of RuNTF. The serum protein binding is known to affect the cytotoxicity, either increasing it or diminishing it, and examples of both can be found in the literature [39,46,48]. The previous results of RuNTF on anti-*T. cruzi* activity [32] indicated that this complex was partially deactivated when incubated in the culture medium, this being ascribed to the binding of RuNTF to proteins (namely BSA, bovine serum albumin) or other components present in the incubation medium. In fact, testing the complex using a plain buffer (PBS-glucose) resulted in a much higher activity. This indicated that protein binding was detrimental for the anti-trypanosomal activity of RuNTF, possibly by affecting its bioavailability (given the fact that the ability to produce nitro anion radicals remained unchanged). In the same work, BSA was taken as a globular protein model and the binding of RuNTF to BSA was investigated, leading to the conclusion that the interaction with BSA occurred very slowly, with the initial 10% binding after two hours becoming 100% binding after 96 h of incubation for a 1:2 protein-to-complex ratio [32]. BSA and HSA share a high degree of homology (over 75% sequence homology) [39] and these prior findings indicated the likelihood of needing to address unusually long contact periods to monitor the HSA-RuNTF interaction. 

This study used CD combined with fluorescence (both steady-state and time-resolved) spectroscopy to access the interaction of RuNTF with HSA. In the UV-Visible range, an ICD(-) band was already detected after 2 h incubation, which is evidence for the formation of an adduct between the protein and RuNTF. This ICD band develops further in intensity upon increasing the complex concentration, thus providing a sound proof of a very close interaction between the compound and the protein, fully consistent with the formation of an {HSA-RuNTF} adduct. This was confirmed by the fluorescence spectroscopy results, with strong quenching of the HSA-Trp214 fluorescence with an increasing complex concentration. 

Our data provides evidence that the interaction of RuNTF with albumin results in the formation of a 1:1 {RuNTF-HSA} adduct with log *K*’_B_ ~4.5 in mimetic physiological conditions. Although the formation of {RuNTF-HSA} was observed for an incubation time as low as 2 h, the equilibrium for this interaction was established only after long contact times (>60 h). The fluorescence data clearly indicate that RuNTF is binding the protein in very close proximity to Trp214, suggesting that the bound RuNTF is located in one of Sudlow’s drug binding sites, with site I being the most likely. The long contact time that is necessary to attain equilibrium suggests that a covalent binding interaction is probably occurring—upon the exchange of one of the more labile ligands (such as chloride) in the RuNTF coordination sphere by a suitable donor from a protein residue. Since this Ru complex binds in very close vicinity of Trp214, the basic N_imidazole_ of His242 is very probable and has been often proposed for other Ru complexes [41,48,49,55]. It is possible that a water molecule is also involved in the stabilization of RuNTF and binding within site I, as found for warfarin and for other molecules [41,56].

The conditional stability constant calculated for the RuNTF-HSA adduct (log *K*’_B_ = 4.5) reveals a moderate to strong interaction that is strong enough to afford a stable adduct while still adequate for a reversible binding. The distribution and delivery through the blood plasma should thus be possible since the value for *K*’_B_({RuNTF-HSA}) is similar to the constant found for Keppler’s complex KP1019 (log *K*’_B_ = 4.0) that proceeded through Phase II Clinical Trials. This was reported to be transported in the blood stream bound to HSA [39,48].

The cytotoxic activity against the A2780 ovarian adenocarcinoma cell line challenged with RuNTF pre-incubated with serum proteins HSA and apo-Tf was evaluated as well. In the case of HSA, the effect of the adduct formation results in a decrease of cell viability of ~20% (at the two highest C_HSA_ tested, 50 and 100 μM) compared to what was determined for the complex tested alone. In the case of apo-Tf, the tendency was the same, and a decrease in cancer cell viability of ~15% was observed at the highest C_Tf_ tested, 30 μM. Overall, this data suggest that the serum protein adducts of RuNTF are at least ~15–20% more active than the Ru complex tested alone, since in the pre-incubation conditions there is probably a mixture of Ru compound alone and RuNTF-HSA. In contrast with cisplatin and its deactivation by binding to serum proteins, our results indicate that RuNTF could use albumin or transferrin as vehicle targeting systems while keeping its activity.

It has been proposed that serum proteins play a role in the mechanism of action of anti-tumoral ruthenium compounds. In the case of KP1019 studies, it has been proposed that HSA may act as a transport device and drug depot, while transferrin has been suggested to be involved in the uptake mechanism [19,39]. The potential importance of these interactions extends further, with the possibility of the formation of protein-complex adducts that either retain their cytotoxic activity against tumor cells or result in cytotoxic adducts per se, taking advantage of a possible passive targeting effect of albumin and transferrin. 

## 4. Materials and Methods

### 4.1. Materials 

All common laboratory chemicals were purchased from commercial sources and used without further purification. The 5-nitrofurylsemicarbazone (L), commercially available in pharmaceutical grade, was purchased from Sigma-Aldrich (St. Louis, MO, USA). Millipore^®^ water was used for the preparation of all aqueous solutions used in the cell-free assays. Phosphate buffered saline (PBS) and fatty acid free human serum albumin (HSA - Sigma Aldrich A3728) were purchased from Sigma-Aldrich. The human *apo*-transferrin (AK3008) was purchased from Akron (Boca Ratón, FL, USA). 

[Ru^II^Cl_2_(dmso)_2_(5-nitrofurylsemicarbazone)], RuNTF, was synthesized from the ruthenium(II) precursor [Ru^II^Cl_2_(dmso)_4_] according to a procedure previously described [33,57]. Shortly, [Ru^II^Cl_2_(dmso)_4_] (100 mg, 0.21 mmol) and L (83 mg, 0.42 mmol) were heated under reflux in ethanol during 8-10 h. A solid precipitated, was filtered off and recrystallized from methanol. 

Yield: 60% (65 mg). Anal. Calc. for C_10_H_18_Cl_2_N_4_O_6_RuS_2_(%): C, 22.9; H, 3.4; N, 10.7; S, 12.2. Found (%): C, 22.8; H, 3.4; N, 10.6; S, 12.3%. FTIR (cm^−1^): 1665 (ν(C=O)); 1533 (ν(C≡N)); 1347 (ν_s_(NO_2_)); 1078 (ν(SO)DMSO). ^1^H-NMR (δ, ppm) (multiplicity, integration, assignment): 3.28 (s, 12H, CH_3_-dmso); 7.70 (d, 1H, nitrofuryl ring); 7.85 (s, 2H, -NH(CO)NH_2_); 7.95 (d, 1H, nitrofuryl ring); 9.34 (s, 1H, -HC=N-); 11.40 (s, 1H, -HC=N-NH-C=O). ^13^C-NMR (δ, ppm) (assignment): 163.9 (-NH-C=O-NH_2_); 152.3, 146.4, 120.3, 114.8 (nitrofuryl ring); 138.8 (-HC=N-); 45.7, 45.8 (CH_3_-dmso) [33].

### 4.2. Physicochemical Characterization 

Furthermore, C, H and N analyses were carried out with a Thermo Scientific Flash 2000 elemental analyzer (Cambridge, UK). The FTIR absorption spectra (4000–300 cm^−1^) of the complex was measured as KBr pellets with a Shimadzu IRPrestige-21 instrument (Kyoto, Japan). The ^1^H- and ^13^C-NMR spectra of the complex were recorded in DMSO-*d*_6_ on a Bruker DPX-400 instrument (at 400 and 100 MHz, respectively, Billerica, MA, USA).

### 4.3. Interaction with Serum Albumin in Cell-Free Media 

The stock solutions of albumin (HSA) were prepared by gently dissolving the protein in pH 7.4 PBS (tablets from Sigma-Aldrich) during a minimum of 60 minutes to allow the protein to fully hydrate. The concentration of each protein stock solution was determined by absorption spectrophotometry using the molar absorption coefficient ε_280_(HSA) = 36,850 M^−1^ cm^−1^ [58].

Given the relative lack of lability inherent to the chemistry of ruthenium compounds, in each essay all spectroscopic measurements were carried out on individually prepared samples to ensure the same pre-incubation time at (37.0 ± 0.1) °C. Due to the limited solubility of the complexes in aqueous media, dimethylsulfoxide (DMSO, from Sigma-Aldrich) was used to prepare the concentrated stock solutions of each complex, followed by appropriate dilution to obtain the desired concentration and the same % of DMSO in the final samples. The DMSO content was kept as 2% (*v/v*) in PBS pH 7.4 buffer in all samples. The dilutions were carried out immediately prior to the samples preparation. 

The UV-Visible isotropic absorption spectra were recorded at room temperature on a Jasco UV-1603 or a Jasco V-560 spectrophotometers (JASCO, Hiroshima, Japan) in the range 270/300–800 nm with 1 cm path quartz Suprasil^®^ cuvettes (Hanau, Germany).

The circular dichroism (CD) spectra were recorded on a JASCO J-720 spectropolarimeter (JASCO, Hiroshima, Japan) with a 175–800 nm photomultiplier (EXEL-308) in the range 300–600 nm and 200–250 nm. The spectra were recorded with quartz Suprasil^®^ CD cuvettes with 1 cm, 0.5 cm or 0.2 cm path length (according to the signal intensity and absorption of samples) at room temperature (ca. 20 °C). Each CD spectrum measured was the result of three to six accumulations originally recorded in ellipticity and converted to Δε = ε_L_ − ε_R_ = *differential absorption* with the Jasco spectropolarimeter software. The CD spectra were represented as Δε_m_ vs. λ with Δε_m_ = {differential absorption}/(*b* C) where *b* is the optical path (cm) and C is the total concentration of the protein (C_HSA_ in Molarity). The samples with a protein concentration of 100 μM (constant) and varying complex concentrations were pre-incubated at (37.0 ± 0.1) °C for ca. 2–62 h prior to measurement in the range 300-600 nm. The samples used to record the CD spectra in the range 200–250 nm were prepared using a protein concentration of 5 μM (constant) and complex concentrations varied from 1:1 to 1:10 protein-to-complex molar ratio and were pre-incubated at (37.0 ± 0.1) °C for ca. 1–68 h at 37 as well. For CD time-dependent assays, the spectra for the 1:2 {protein}:{RuNTF} ratio were recorded at (32 ± 2) °C using a water bath equipped with a temperature control, with the samples being kept at the same temperature in-between the measurements (from 1 h to 68 h incubation).

The fluorescence measurements were carried out on a Fluorolog 3.22 spectrofluorometer from Horiba Jobin Yvon (Palaiseau, France) at (25.0 ± 0.1) °C using a water bath equipped with a temperature control. For these measurements, the final protein concentration in the (individually prepared) samples was 5 μM, and the complex concentration varied accordingly to obtain HSA:Ru-complex molar ratios ranging from 1:1 to 1:30. The samples with the same complex concentration but with no protein were prepared for the appropriate background correction.

For steady-state fluorescence intensity measurements, the excitation was at 295 nm. In all Stern-Volmer plots, the values were corrected for the absorption and emission inner filter effects [53,59] using the absorbance recorded for each sample at the relevant wavelengths. The bandwidth was typically 4 nm for both the excitation and emission. A nanoLED N-280 (Horiba Jobin Yvon) was used for the excitation of HSA for the time-resolved measurements by the single photon counting technique, and the emission was collected at 350 nm with a 10 nm emission bandwidth. This wavelength was chosen because it is near the maximum emission wavelength (so the sensitivity is close to maximum), but it is further to the red in relation to the water Raman scattering peak. Therefore, the signal-to-background ratio is much more favorable to obtain good quality data than it would be if the actual maximum emission wavelength (334 nm) was used. 

The experimental fluorescence intensity decays were analyzed by fitting a sum of exponentials according to:

(4) $$I\left(t\right)=\;\overset{n}{\underset{i=1}{\sum }}p_{i}\;\exp(-t/\tau_{i})$$

where *p*_i_ and τ_i_ are the pre-exponential factors and lifetime of component *i*, respectively, using the TRFA Data Processor program (version 1.4; Minsk, Belarus). The criteria for assessing the quality of the fit were a reduced χ^2^ value close to 1 and the random distribution of weighted residuals and the residuals autocorrelation. The amplitude of each lifetime component *α_i_* is the normalized pre-exponential (*p_i_*/Σ*_i_* *p_i_*) such as Σ*_i_* *α_i_* = 1. The intensity-weighted mean fluorescence lifetime, i.e., the mean lifetime of the excited singlet state, was obtained through Equation (5),

(5) $$\langle \tau \rangle \;=\;\overset{n}{\underset{i=1}{\sum }}\alpha_{i}\tau_{i}^{2}/\overset{n}{\underset{i=1}{\sum }}\alpha_{i}\tau_{i}\;\;\;$$

The amplitude-weighted mean fluorescence lifetime is given by:

(6) $$\bar{\tau }=\;\overset{n}{\underset{i=1}{\sum }}\alpha_{i}\tau_{i}$$

and allows the evaluation of the changes in the quantum yield by processes affecting the fluorescence intensity decay profile. Ludox^®^ (Sigma-Aldrich, TM-50 colloidal silica, 50 wt. % suspension in water) was used to obtain the instrumental response function.

### 4.4. Cell Assays–Cytotoxicity Assessment

The cytotoxic activity of RuNTF was evaluated in human cancer cells A2780 ovarian (Sigma-Aldrich), MCF7 breast (ATCC) and PC3 prostate (ATCC) and a non-tumoral V79 lung fibroblasts (ATCC). The cell lines were grown in RPMI (A2780 and PC3) and GlutaMAX™-I. (MCF7 and V79) medium supplemented with 10% FBS and maintained in an incubator (Heraeus, Germany) in a humidified atmosphere at 37 °C and 5% CO_2_.

The cell viability was measured by the MTT (3-[4,5-dimethylthiazol-2-yl]-2,5 diphenyl tetrazolium bromide) assay, based on the conversion of the tetrazolium bromide into formazan crystals by metabolic active cell [60]. For the assay, the cells were seeded in 96-well plates at a density of 1 × 10^4^–2 × 10^4^ cells in 200 μL medium and allowed to attach for 24 h. The complex was first diluted in DMSO to dissolve, and then in a medium to prepare the serial dilutions in the range 10^−7^–10^−4^ M. The maximum concentration of DMSO in the medium was 1% and had no cytotoxic effect. After careful removal of the medium, 200 μL of each dilution was added to the cells and incubated for 72 h at 37 °C. At the end of the incubation, the medium was discarded and 200 μL of MTT solution in PBS (1.5 mM) was added to each well. After 3 h at 37 °C, the medium was discarded and 200 μL of DMSO was added to solubilize the formazan crystals. The cellular viability was assessed measuring the absorbance at 570 nm using a plate spectrophotometer (Power Wave Xs, Bio-Tek, Winooski, VT, USA). The IC_50_ values were calculated from the concentration-response plots using the GraphPad Prism software (version 5.0). The results are the mean ± standard deviation (SD) of the two independent experiments done with six replicates each. 

### 4.5. Effect of Serum Protein Binding in the Cytotoxicity against Human Tumour Cells

Albumin (fatty acid free) and human apo-transferrin solutions were prepared in PBS following a previous described method to obtain the desired protein/complex molar ratios [61,62]. The protein concentration was assessed by UV spectrophotometry using ε_280_(HSA) = 36,850 M^−1^cm^−1^ for HSA and ε_280_(apoTf) = 92,300 M^−1^cm^−1^ [58]. The effect of HSA on the cytotoxic activity of the complex was evaluated for a 20 µM complex concentration (in HSA measurements) and a 5 µM complex concentration (in Tf measurements). The protein solutions were pre-incubated for 2 h at 37 °C to form the complex/protein adducts. During incubation for 72 h with A2780 cells, the medium supplement fetal bovine serum was reduced to 5% [63,64]. The MTT assay was performed as described above. The results are the mean ± standard deviation (SD) of two independent experiments with six replicates each, and the IC_50_ values were calculated using the GraphPad Prism software (as above).

## Supplementary Materials

The following are available online at https://www.mdpi.com/1420-3049/24/16/2861/s1, Figure S1: UV-Vis spectroscopy for the system RuNTF—HSA over time; Figure S2: CD spectra obtained for the system HSA–NTF ligand; Figure S3: Far-UV CD spectrum for the system RuNTF—HSA over time; Figure S4: Effect of increasing concentrations of RuNTF on the secondary structure of HSA. Figure S5: Cytotoxicity of RuNTF complex against non-cancer cell lines.
