# Supplementary material for: Interaction with Blood Proteins of a Ruthenium(II) Nitrofuryl Semicarbazone Complex: Effect on the Antitumoral Activity
Source: Molecules. 2019 Aug 7;24(16):2861. doi: 10.3390/molecules24162861 (PMC6719144; doi:10.3390/molecules24162861)
Supplement: Supplementary file 1 [file molecules-24-02861-s001.pdf]

Supplementary Material

Interaction with Blood Proteins of a Ruthenium(II) Nitrofuryl Semicarbazone Complex: Effect on the Antitumoral Activity

Bruno Demoro [1], Andreia Bento-Oliveira [2,3], Fernanda Marques [4], João Costa Pessoa [5], Lucía Otero [1], Dinorah Gambino [1,*], Rodrigo. F. M. de Almeida [2,3] and Ana Isabel Tomaz [2,*]

1 Cátedra de Química Inorgánica, Facultad de Química, Universidad de la República – UDELAR, 11800 Montevideo, Uruguay
2 Centro de Química Estrutural, Faculdade de Ciências – CQE-FCUL, Universidade de Lisboa, 1749-016 Lisbon, Portugal
3 Centro de Química e Bioquímica, Faculdade de Ciências da Universidade de Lisboa, 1749-016 Lisbon, Portugal
4 Centro de Ciências Tecnológicas e Nucleares, Instituto Superior Técnico – C[2]TN-Técnico, Universidade de Lisboa, Campus Tecnológico e Nuclear, 2686-953 Sacavém, Portugal
5 Centro de Química Estrutural, Departamento de Engenharia Química, Instituto Superior Técnico, Universidade de Lisboa, 1049-001 Lisbon, Portugal
* Correspondence: dgambino@fq.edu.uy (D.G.); isabel.tomaz@ciencias.ulisboa.pt (A.I.T.)

Commented [m1]: Please carefully check the accuracy of names and affiliations. Changes will not be possible after proofreading.

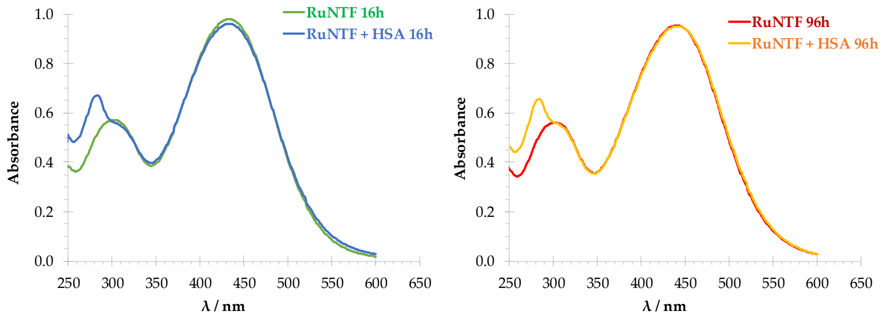

(a) (b)

Figure S1. UV-Vis spectroscopy for the system RuNTF—HSA over time: isotropic absorption spectra of solutions of RuNTF complex in the absence (green, red) and in the presence (blue, orange) of HSA after **(a)** 16h and **(b)** 96 h of incubation at 37°C [in 2%DMSO/PBS buffer, spectra recorded at room temperature (20.0 ± 0.5)°C, C_{RuNTF} = 60 μM)].

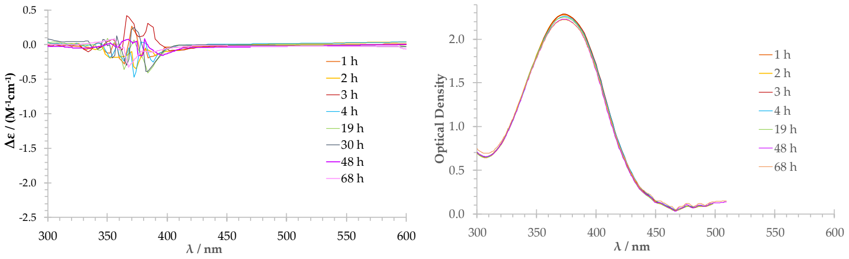

(a) (b)

Figure S2. UV-Vis spectroscopy for the system L—HSA over time: (a) CD spectra of solutions of the 5-nitro-2-furaldehyde semicarbazone ligand L and HSA; and **(b)** their corresponding isotropic absorption spectra; the incubation time at 37°C is indicated. [Conditions: 1%DMSO/PBS buffer, spectra recorded at room temperature (20.0 ± 0.5)°C, $C_{HSA} = C_{RuNTF} = 200$ μM)].

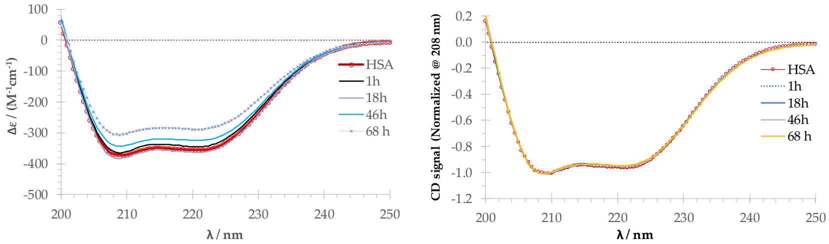

(a) (b)

Figure S3. Far-UV CD spectrum for the system RuNTF—HSA over time: (a) Far-UV CD spectrum of solutions containing HSA (red, 5 μM) and HSA-RuNTF at a 1:4 molar ratio (blue) with increasing incubation time; **(b)** CD signal intensity normalized at $\lambda^{(-)}_{max}$ = 208 nm for HSA-RuNTF 1:4 molar ratio recorded at increasing incubation times up to 68h **[**C_{HSA}= 100 μM = constant; C_{RuNTF}: 20 μM; samples in PBS pH7.4 incubated at 37°C; spectra recorded at room temperature (20.0 ± 0.5)°C].

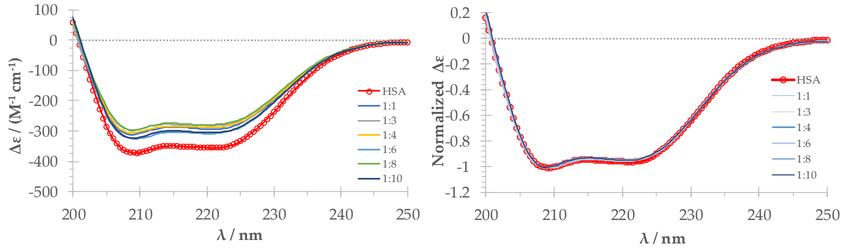

(a) (b)

Figure S4. Effect of increasing concentrations of RuNTF on the secondary structure of HSA: (a) Far-UV spectra of HSA in the presence (colored lines) and absence (red line, open circles) of increasing concentrations of RuNTF complex following a 68h incubation period **(b)** CD spectra with Δε values

normalized at $\lambda^{(-)}_{max}$=208 nm [C_{HSA}= 5 µM, kept constant; C_{RuNTF}: 0-50 µM; samples in PBS pH7.4 incubation at 37°C; spectra recorded at room temperature (20.0 ± 0.5)°C.].

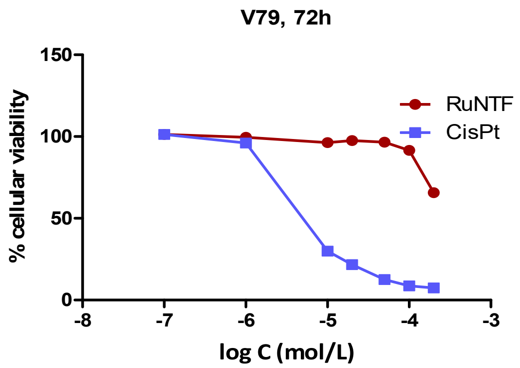

Figure S5. Cytotoxicity of RuNTF complex against non-cancer cell lines: dose-response curve obtained for the determination of the IC50 value in the V79 lung fibroblasts upon 72h incubation with RuNTF and cisplatin (CisPt). (IC_{50} > 200 µM; results are average values from 2 independent experiments with a minimum of 6 replicates.)
